# Reviewed article: Santos ME, Ferreira RC, Pinheiro EL, Vieira SSA, Senna MIB. Organizational readiness for change and work process of oral health teams in Primary Health Care: an exploratory study in the initial implementation phase of MonitoraSB, Minas Gerais, Brazil, 2024. Epidemiol Serv Saude. 2025:34;e20240802

**DOI:** 10.1590/S2237-96222025v34e20240802.a

**Published:** 2025-10-20

**Authors:** Roberta Jorge

**Affiliations:** 1 Universidade do Estado do Rio de Janeiro, Rio de Janeiro, RJ, Brazil

## Peer review A

## Questionnaire

Does the manuscript contain new and significant information to justify publication? Yes

Does the Abstract (Summary) clearly and accurately describe the content of the article? No

Is the problem significant and concisely stated? Yes

Are the methods described comprehensively? No

Are the interpretations and conclusions justified by the results? No

Is adequate reference made to other work in the field? Yes

## Manuscript Structure

Length of article is: Adequate

Number of tables is: Adequate

Number of figures is: Adequate

Please state any conflict(s) of interest that you have in relation to the review of this paper. None

Interest: Good

Quality: Good

Originality: Excellent

Overall: Good

Recommendation: Major Revision

## Comments

Prezados autores, parabéns pelo estudo! A temática é relevante e pertinente. Considero que ajustes são necessários e descrevo-os abaixo.

REFERÊNCIAS:

Considero que um artigo deve ser citado por vocês. Aborda o MonitoraSB e pode sustentar diversos pontos cujas informações carecem de maior aprofundamento, como a justificativa para o monitoramento e avaliações dos serviços. Referência: Ferreira RC, Houri LCLF, Amaral JHLD, Santos ME, Pinheiro EL, Gomes PM, Oliveira RMM, Prado RLD, Rocha NBD, Pereira HB, Santos JS, Cruz DSD, Senna MIB. MonitoraSB: an innovation for monitoring and strengthening oral health in primary health care in Brazil. Rev Bras Epidemiol. 2024 Dec16;27:e240065. doi: 10.1590/1980-549720240065. PMID: 39699462; PMCID: PMC11654291.

